# Increased amygdala reactivity following early life stress: a potential resilience enhancer role

**DOI:** 10.1186/s12888-017-1201-x

**Published:** 2017-01-18

**Authors:** Tetsuya Yamamoto, Shigeru Toki, Greg J. Siegle, Masahiro Takamura, Yoshiyuki Takaishi, Shinpei Yoshimura, Go Okada, Tomoya Matsumoto, Takashi Nakao, Hiroyuki Muranaka, Yumiko Kaseda, Tsuneji Murakami, Yasumasa Okamoto, Shigeto Yamawaki

**Affiliations:** 1 Department of Psychiatry, University of Pittsburgh School of Medicine, 121 Meyran Avenue, Loeffler Building, 15260-5003 Pittsburgh, PA USA; 2 Japan Society for the Promotion of Science, 8 Ichiban-cho, Chiyoda-ku, Tokyo, 102-8472 Japan; 3 Department of Psychiatry and Neurosciences, Institute of Biomedical and Health Sciences, Hiroshima University, 1-2-3 Kasumi, Minami-ku, 734-8551 Hiroshima, Japan; 4 Western Psychiatric Institute and Clinic, 3811 O Hara St, 15213-2593 Pittsburgh, PA USA; 5 Faculty of Psychology, Otemon Gakuin University, 2-1-15 Nishiai, 567-8502 Ibaraki, Osaka Japan; 6 Department of Psychology, Graduate School of Education, Hiroshima University, 1-1-1 Kagamiyama, 739-8524 Higashi-Hiroshima, Hiroshima Japan; 7 Faculty of Health Sciences, Tsukuba International University, 6-20-1 Manabe, 300-0051 Tsuchiura, Ibaraki Japan; 8 Department of Radiology, Hiroshima City General Rehabilitation Center, 1-39-1 Tomo-minami, Asaminami-ku, 731-3168 Hiroshima, Japan; 9 Kure Kyosai Hospital, 2-3-28 Nishi-chuo, 737-8505 Kure, Hiroshima Japan; 10 Present address. Graduate School of Integrated Arts and Sciences, Tokushima University 1-1, Minamijosanjima-cho, 770-8502 Tokushima, Japan

**Keywords:** Early life stress, Amygdala reactivity, fMRI, Resilience, Depression

## Abstract

**Background:**

Amygdala hyper-reactivity is sometimes assumed to be a vulnerability factor that predates depression; however, in healthy people, who experience early life stress but do not become depressed, it may represent a resilience mechanism. We aimed to test these hypothesis examining whether increased amygdala activity in association with a history of early life stress (ELS) was negatively or positively associated with depressive symptoms and impact of negative life event stress in never-depressed adults.

**Methods:**

Twenty-four healthy participants completed an individually tailored negative mood induction task during functional magnetic resonance imaging (fMRI) assessment along with evaluation of ELS.

**Results:**

Mood change and amygdala reactivity were increased in never-depressed participants who reported ELS compared to participants who reported no ELS. Yet, increased amygdala reactivity lowered effects of ELS on depressive symptoms and negative life events stress. Amygdala reactivity also had positive functional connectivity with the bilateral DLPFC, motor cortex and striatum in people with ELS during sad memory recall.

**Conclusions:**

Increased amygdala activity in those with ELS was associated with decreased symptoms and increased neural features, consistent with emotion regulation, suggesting that preservation of robust amygdala reactions may reflect a stress buffering or resilience enhancing factor against depression and negative stressful events.

## Background

Increased reactivity to emotional information is characteristic of depression, and has been linked with increased and sustained reactivity in the amygdala [1–4]. Hyper-reactivity is often associated with vulnerability to depression as it occurs in populations that tend to become depressed such as children with anxiety or depressed parents [5] as well as those at risk for depressive relapse [6], those with early life stress (ELS) [7], cognitively vulnerable individuals [8], and individuals with inhibited temperament [9]. That said, not all individuals with high levels of vulnerability become depressed. Rather, we will consider whether amygdala hyper-reactivity, as a consequence of early stress, may contribute to resilience against developing depression in otherwise vulnerable individuals. This is important as intervening on hyper-reactivity prior to the onset of depression would be either indicated or contra-indicated based on its causal role.

A specific vulnerability factor for depression, history of ELS, has been linked to both amygdala hyper-reactivity [10] and hypo-reactivity [11]. Effects of stress on the amygdala [12] are hypothesized to underlie alterations in cognition, mood, and behavior [13–15]. These changes have been further hypothesized to shape individual differences in vulnerability for mood and anxiety disorder, such as emotional reactivity [16–18]. That said, there is scant evidence for this complete causal pathway.

Rather, a great deal of data shows that early life stressors are associated with increased amygdala reactivity in the absence of psychiatric diagnoses [15]. This could represent vulnerability for future depression, or could suggest that neural adaptations to stress are protective.

In fact, decreased amygdala activity may be a vulnerability factor. For example, patients with borderline personality disorder are vulnerable to depression and frequently display decreased amygdala activity during emotional challenges [19]. The capacity to react to emotional information is hypothesized to be protective with blunted reactivity being more clearly associated with pathology [20] including depression [21].

Here we suggest that in vulnerable individuals, robust amygdala reactivity may be protective compared to more blunted amygdala reactivity. Adaptive responses to stressors in childhood can have a “stress inoculating” effect, and lead to resilience to future stressors [22]. To clarify the role of amygdala reactivity in resilience, we examined, using functional magnetic resonance imaging (fMRI), whether increased amygdala activity down-modulated depressive symptoms and the impact of life events in individuals with a history of ELS but no history of depression. Since our goal was to examine the extent to which ELS might confer vulnerability for future depression via increasing depressive severity within a subclinical range, we recruited healthy people who did not have severe depressive symptoms. Subclinical depressive severity was interpreted as an index of vulnerability [23]. Also, to examine potential mechanisms for preserved reactivity we further explored functional connectivity with the amygdala in this sample.

## Methods

### Participants

Thirty healthy volunteers, who did not have any physical and mental problems, were recruited through adverts in local newspapers and public notices. All participants were interviewed by a psychiatrist or psychologist to assess for a health condition and a lifetime history of DSM-IV psychiatric disorders using the MINI International Neuropsychiatric Interview [24, 25]. Exclusion criteria were as follows: being left-handed or ambidextrous [26], history of seizures, head trauma, use of psychotropic medications, magnetic resonance imaging (MRI) contraindications or technical alterations, unsuccessful induction of negative mood and participants who exceed the cutoff score for depressive symptoms (Beck Depression Inventory II >29) [27, 28]. All participants scored in the normal range on a cognitive screen [29, 30], with a verbal IQ-equivalent >80. We excluded three participants in whom the negative mood induction failed to produce an increase in negative mood or decrease in positive mood, two who exceeded the cutoff score for depressive symptoms [27, 28], and one who showed excessive head movement (>3.0 mm over the functional MRI run). Thus, 24 participants (6 male) were included in the analyses. One participant was missing fMRI data from one rest block. As such, their data was included in analyses of block-related averages but not in time-series analyses for which complete data were required.

The study was approved by the Research Ethics Committee of Hiroshima University. After complete description of the study to the participants, written informed consent was obtained. Participants received 5,000 Japanese yen (~US$42) to compensate them for their time.

### Self-report Measures

We assessed early life stress (ELS) using the Japanese version of the Child Abuse and Trauma Scale (CATS) [31, 32]. The CATS is a 38-item retrospective self-report questionnaire that measures subjective perception of four ELS subtypes (negative home environment/neglect, sexual abuse, punishment, and emotional abuse). Participants rated how frequently they experienced particular adverse events experience during their childhood and adolescence using a five–point scale (0 = never, 4 = always). Scores for each factor are calculated based on the mean value of the individual items for each subscale, and range between 0 and 4. Higher mean values represent more severe ELS. The CATS has favorable psychometric properties, including adequate test-retest reliability, internal consistency, and concurrent validity [31, 33]. As shown in Table 1, participants reported similar CATS scores to those in a previous study that used healthy undergraduate students [31]. Scores generally fell within what is described by the measure’s authors as the mild to moderate range.

**Table 1:** Demographic and Subjective Data

Measure	Mean	(SD)	[Range]
Age (years)	40.70	(11.05)	[24-60]
Verbal Intelligence	110.36	(6.62)	[97-120]
PHQ-9	3.67	(3.78)	[0-14]
CATS Total Score	0.84	(0.60)	[0.2-2.5]
Negative Home Environment/Neglect	0.85	(0.64)	[0-2.8]
Sexual Abuse	0.10	(0.18)	[0-.0.7]
Punishment	1.51	(0.74)	[0-2.7]
Emotional Abuse	0.89	(0.85)	[0-2.9]
LES Impact of Negative Life Event	2.58	(4.86)	[0-23]
Difficulty to recall sad memory	3.21	(2.77)	[0-9]
Vividness of recalled sad memory	6.08	(2.87)	[1–10]

*CATS* Childhood Abuse and Trauma Scale, *LES* Life Experiences Survey, *PHQ-9* Patient Health Questionnaire-9

**Fig. 1:**
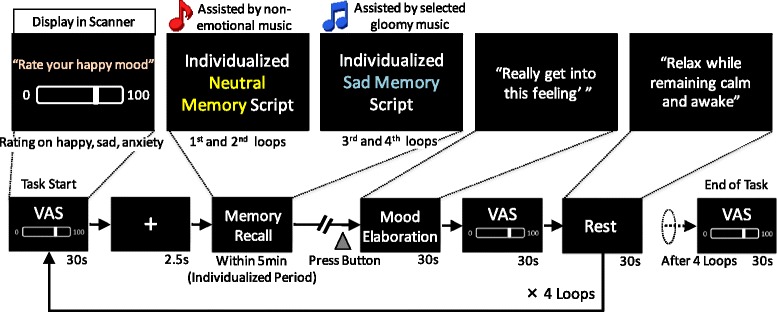
Procedure of modified version of mood induction paradigm. VAS, Visual Analogue Scale

Participants’ mood states during the scanning session were assessed using a computer based Visual Analogue Scale (VAS) (Fig. 1). Participants rated their moods on three unipolar VAS measuring happiness, sadness, and anxiety dimensions by moving a cursor with a four button response pad. The VAS dimensions were projected onto a screen in the MR scanner. The scales ranged from 0 to 100, with 0 indicating “not at all” and 100 indicating “extremely”. In a debriefing session after scanning, we asked participants to rate the extent to which they were successfully engaged in the mood induction task (which required recall of personal experiences) on two 11-point scales (0–10). Participants rated “difficulty to recall memory,” and “vividness of the recalled memories.”

We assessed the impact of recent negative life events using the Japanese version of the Life Event Stress Scale (LES) [34, 35]. For the LES, participants were asked to indicate which of 57 events had occurred during the previous 12 months, and to rate the impact of each event using a seven-point scale, ranging from extremely negative (−3) to extremely positive (+3). The total scores for impact of negative life events were used.

Current symptoms of depression were assessed using the Japanese version of the Patient Health Questionnaire-9 (PHQ-9) [36, 37]. Verbal intelligence was estimated using the Japanese National Adult Reading Test [29, 30].

### Mood induction paradigm

To develop an individualized, negative mood inducing fMRI task, we used a combination of re-experiencing personal emotional episodes and listening to music associated with sad mood. We modified a mood induction paradigm developed by Ramel et al. [6] and Segal et al. [38], which was used in an individualized version of a block design paradigm.

For the memory recall procedure, participants were asked to write four detailed autobiographical scripts about two very sad personal experience (sad memory) and two specific but unemotional days in their lives (neutral memory). On a scale from 1 (neutral) to 9 (extremely sad), they were encouraged to describe sad episodes that they rated 5 or higher. For the neutral memory, participants were also asked to write in detail about a specific but unemotional day in their lives. The scripts were sent to participants about one week prior to the session day.

In a pre-scanning session, participants were asked to listen to the first minute of four music pieces on a PC. Using a computer-based VAS, they rated (a) the degree to which the music created a sad impression and (b) the degree to which the music was able to bring about sadness. The VAS ranged from 0 to 100, with 0 indicating “not at all” and 100 indicating “extremely”. The music consisted of a standard selection battery [39], which has been used in multiple mood induction studies [6, 40, 41]. These included: *Russia under the Mongolian Yoke* composed by Sergei Prokefiev, played at half speed; *Adagio for Strings* composed by Samuel Barber; *Peer Gynt - The Death of Ase,* composed by Edvard Grieg; and *Adagio in G Minor* composed by Tomaso Albinoni. The sad memory recall procedure was accompanied by the two sad music selections that were rated most highly on each VAS for each individual. The neutral recall condition was accompanied by the musical pieces *Venus, the Bringer of Peace* and *Neptune, the Mystic* by Gustav Holst.

To ensure that an appropriate mood was induced in participants, we used an altered block design paradigm (Fig. 1) in which a standardized 30 s block from the same emotional set of stimuli commenced only after the participant indicated (via a button press) that they were clearly experiencing a neutral or sad mood. While in the MR scanner, participants listened to the selected musical piece (presented via headphones) while reading and attempting to re-experience the sadness of the event depicted in the autobiographical script, which was projected onto a screen. Each musical piece and autobiographical script was presented for up to 5 min (an individualized recall period) before each of the 30 s blocks. The duration of presentation depended on the time it took for the participant to achieve the appropriate mood state. Music was not played during the 30 s block periods.

All participants were exposed to eight 30 s alternating blocks of recall (sad or neutral) followed by resting periods, in addition to the individualized recall period. Participants’ mood state was assessed using a VAS during the scanning session. Participants were first asked to recall an emotionally neutral memory and then a sad memory. On the basis of a previous study [42], this design was adopted to avoid contamination of the neutral stimuli by the sad stimuli.

### fMRI image acquisition

Images were acquired using a 3.0 T MRI scanner (SIGNA HDxt; GE; single-shot, echo planar imaging (EPI) with whole-brain coverage, 32 axial slices per 2500 ms TR, TE = 30 ms, flip angle = 90°, matrix size = 64 × 64, FOV = 240 mm, slice thickness = 4 mm, inter-slice gap = 0 mm). A high resolution T1-weighted image provided anatomical localization (Ir-P FSPGR; TE = 1.9 ms, TR = 6.9 ms, flip angle = 20°, matrix size = 256 × 256, FOV = 25.6 mm, slice thickness = 1 mm, inter slice gap = 0 mm, 180 slices).

### fMRI data preprocessing

Preprocessing and analysis of fMRI data were conducted using the statistical parametric mapping software package, SPM 8 (Wellcome Department of Cognitive Neurology, London, UK). The first 4 volumes of the fMRI run were discarded to ensure a steady-state MR signal. Time-series were slice-time corrected, volume registered to the mean image, and coregistered with T1-weighted structural images. T1 images were bias-corrected and segmented using the International Consortium for Brain Mapping (ICBM) tissue probability maps for gray matter, white matter, and cerebrospinal fluid. Time-series data were spatially normalized to the ICBM152 template, smoothed with an 8 mm FWHM Gaussian kernel, and high-pass filtered at 0.008 Hz.

### Behavioral data analyses

To test whether participants showed both greater increases in sadness and greater reductions in happiness after sad memory recall than after neutral memory recall, we used a two-way repeated measures analysis of variance (ANOVA). We used two within-subject factors (mood: happy, sad, and anxious; time: baseline /VAS1, post-1^st^ neutral memory recall [NR] /VAS2, post-1^st^ rest/VAS3, post-2^nd^ NR/VAS4, post-2^nd^ rest/VAS5, post-1^st^ sad memory recall [SR] /VAS6, post-3^rd^ rest/VAS7, post-2^nd^ SR/VAS8, and post-4^th^ rest/VAS9). To control for Type I errors across the analyses, we used the Bonferroni procedure. Significance level was set at *p* < 0.05. One participant was excluded from the analysis due to missing data following a scanner problem.

To examine the relationships between mood change, ELS, depression, the impact of recent negative life events, and the degree of task engagement, we conducted a Pearson’s correlation analysis. Mood change (the effect of mood induction on mood) was operationalized by comparing mood after sad memory recall with mood after neutral memory recall, i.e., mood change = ([VAS6 + VAS8] – [VAS2 + VAS4])/2. Significance level was set at *p* < 0.05 (two-tailed).

All behavioral analyses were performed using SPSS v. 22.0 (SPSS Japan Inc., Tokyo, Japan).

### fMRI data analyses

To visualize the amygdala region activated during sad memory recall, preprocessed time series data for each participant were analyzed using multiple regression. We measured amygdala activity during the sad/neutral mood elaboration period (30 s; Fig. 1). The model included a regressor for the contrast term ‘sad mood recall vs. neutral mood recall’. Thus, for each voxel, amygdala reactivity = BOLD_Sad Recall_ – BOLD_Neutral Recall_. Given our focus on amygdala reactivity, amygdala activation was examined using small volume correction. We used a statistical threshold of *p* < 0.05, family wise error (FWE) corrected, for the bilateral amygdala with an extent threshold of 10 contiguous voxels. The amygdala was defined according to Tzourio-Mazoyer et al. [43], and one bilateral amygdala mask was created using the WFU PickAtlas [44]. In a second step, the mean contrast values for the significant cluster from the initial analysis were extracted using the MarsBaR region of interest (ROI) toolbox (version 0.43) [45], and further analyzed using SPSS 22 (SPSS Japan Inc., Tokyo, Japan). Subsequent analyses were performed using mean contrast values, except for two analyses of the time-series of amygdala activity and the generalized psychophysiological interaction (gPPI) analysis.

We conducted Pearson’s correlation analyses using each of the four CATS subscale scores and amygdala activity, to examine the association of history of ELS and amygdala responsiveness. We also evaluated a hierarchical multiple regression model predicting amygdala reactivity using the CATS scores, age, PHQ-9 scores, LES negative life events scores, and task engagement (difficulty recalling a memory and the vividness of the recalled memory). Significance for correlations was set at *p* < 0.05 (two-tailed).

We also investigated the differences in the time-series of amygdala activity between people with a past history of ELS and people with no history. Activity in the significant clusters within the amygdala, derived from the mood induction task, were extracted and averaged using the 12 scans (30 s) preceding each VAS rating during the mood elaboration period. Activation in the amygdala was expressed as a percentage difference from a pre-stimulus baseline (VAS1). We performed a three-way repeated measures ANOVA with one between-subject factor (group: ELS and non-ELS) and two within-subject factors (mood; time). One non-ELS participant was excluded due to missing data following a scanner problem.

Finally, to examine the moderated effect of amygdala reactivity on depressive symptoms and the impact of negative life events in people with a history of ELS, we performed moderated multiple regression involving ELS as a predictor, amygdala reactivity as a moderator, and depression and impact of negative life events as dependent variables. To capture the way in which activity in other brain regions modulates amygdala reactivity, we used the gPPI toolbox [46]. The seed regions were significant clusters of activity in the bilateral amygdala that were identified in the preceding analysis for mood induction. As a task regressor, we used the contrast term ‘sad mood recall – neutral mood recall’. The obtained individual gPPI images were used to perform a random effect analysis using a whole-brain two sampled t-test. The threshold of the gPPI analysis was set at *p* <0.005, uncorrected. Cluster k extent, determined by 1000 Monte Carlo simulations at the whole-brain level, was implemented in AlphaSim [47] for 290 voxels (for the left amygdala seed) and 253 voxels (for the right amygdala seed), and at cluster levels of *p* < 0.05, corrected.

Each statistical threshold of fMRI data analysis was set on the basis of previous studies [10, 11, 48].

## Results

### Mood manipulation check

**Fig. 2:**
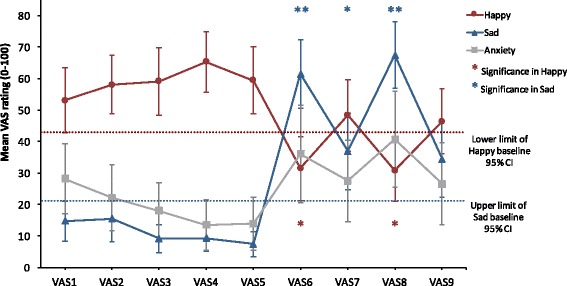
Mood induction effects on mood. *Error bars* represent the 95% confidence interval (CI). *,**, Significant difference from VAS1 (baseline) score (*p < 0.05, **p < 0.001, two-tailed); VAS1, baseline; VAS2, post-1^st^ neutral memory recall (NR); VAS3, post-1^st^ rest; VAS4, post-2^nd^ NR; VAS5, post-2^nd^ rest; VAS6, post-1^st^ sad memory recall (SR); VAS7, post-3^rd^ rest; VAS8, post-2^nd^ SR; VAS9, post-4^th^ rest

Mood × time repeated measures ANOVA revealed a significant mood × time interaction, Greenhouse-Geisser *F* (3.79, 83.35) = 24.73, *p* <. 001, *η*_p_^2^ = .53. Paired samples t-tests of change over time within conditions showed that after sad memory recall (VAS6 and VAS8), participants demonstrated significantly increased sadness ratings, and significantly decreased happiness ratings in comparison with baseline (VAS1) and before sad memory recall (VAS2, VAS3, VAS4, and VAS5), while anxiety ratings remained constant throughout the experiment (Fig. 2). Most of the participants were able to recall vividly both neutral memory and sad memory (Table 1). While some people reported the difficulty of recalling memory, we confirmed that their moods were successfully manipulated.

### Relationship between early life stress, mood change and amygdala reactivity

#### Mood change

There was a non-significant positive correlation between sad mood change and CATS sexual abuse scores, *r* = .38, *p* = .071, and a significant negative correlation between happy mood change and CATS sexual abuse, *r* = -.51, *p* = .014. There were no associations of mood change and other self-report measures, all *r*s < -.20, *p*s > .368.

#### Amygdala reactivity

There were significant bilateral amygdala regions for the sad vs neutral mood contrast, left amygdala, x = -28, y = -4, z = -22, *Z* = 3.65, *p*_FWE-corrected_ = .001, k = 34, and right amygdala, x = 28, y = -4, z = -20. *Z* = 3.92, *p*_FWE-corrected_ < .001, k = 68.

CATS sexual abuse scores were significantly correlated with right amygdala activity, *r* = .48, *p* = .018. There was a non-significant correlation between CATS sexual abuse and left amygdala activity, *r* = .38, *p* = .064. The other CATS subscales did not significantly correlate with amygdala activity, all *r*s < -.27, *p*s > .195.

Hierarchical multiple regression analysis showed that sexual abuse scores significantly predicted right amygdala reactivity above and beyond other features, *ΔF* (1, 18) = 9.81, *ΔR* ^2^ = .29, *p* = .006, *β* = .61, and for the left amygdala reactivity, *ΔF* (1, 18) = 5.65, *ΔR* ^2^ = .22, *p* = .029, *β* = .53. No other CATS subscale had significant effects, all *ΔF*s < 1.82, *p*s > .195.

### Influences of early life stress on time-series of mood change and amygdala activity

Based on these results, additional analyses further addressed whether increased amygdala reactivity was likely to be protective or a vulnerability factor within the child sexual abuse group. We divided the participants into two groups: those who had reported child sexual abuse (*n* = 7, 0 male; ELS group) and those without reported child sexual abuse (*n* = 17, 17 male; non-ELS group). There were moderately strong but non-significant differences between these groups including age, *t* (22) = 1.02, *p* = .32, *d* = .46, sex, *χ* ^2^ (1) = 3.29, *p* = .13, *V* = .36, xdepressive symptoms, *t* (7.78) = 1.10, *p* = .30, *d* = .62, negative life events, *t* (6.45) = 1.32, *p* = .23, *d* = .87, vividness of recalled memory, *t* (22) = 1.88, *p* =. 073, *d* = .84, and difficulty of memory recall, *t* (22) = 1.59, *p* = .127, *d* = .71.

#### Time-series of mood

**Fig. 3:**
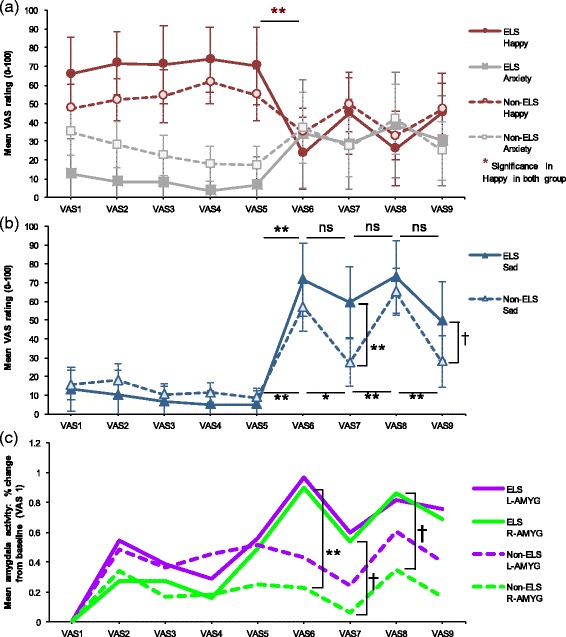
Relationship among experience of early life stress, mood change, and amygdala activity. **a** Mood induction effects on happiness and anxiety in ESL group and non-ESL group. **b** Mood induction effects on sadness in ESL group and non-ESL group. **c** Time courses for right and left amygdala activity in significant cluster. *Error bars* represent the 95% confidence interval. †,*,**, Significant difference for a priori time points of interest (†*p* < 0.10, *p < 0.05, **p < 0.01, two-tailed). VAS1, baseline; VAS2, post-1^st^ neutral memory recall (NR); VAS3, post-1^st^ rest; VAS4, post-2^nd^ NR; VAS5, post-2^nd^ rest; VAS6, post-1^st^ sad memory recall (SR); VAS7, post-3^rd^ rest; VAS8, post-2^nd^ SR; VAS9, post-4^th^ rest; ESL, early life stress; L-AMYG, left amygdala; R-AMYG, right amygdala

Group (ELS and non-ELS) × Mood × Time repeated measures ANOVA revealed a significant three-way interaction, Greenhouse-Geisser *F* (4.40, 92.33) = 3.47, *p* = .009, *η*_p_^2^ = .14. As with Fig. 2, both groups demonstrated significantly increased sadness ratings, and significantly decreased happiness ratings after sad memory recall (VAS6 and VAS8; Fig. 3a, b). There were not any significant group differences for the happiness and anxiety at each time point, and these groups reported consistent changes across the experiment (Fig. 3a). However, ELS participants did not show significant decreases in sadness at the rest period after sad memory recall (VAS7 and VAS9) in comparison with the prior time point (VAS6 and VAS8), while non-ELS participants showed significantly decreased sadness at the same time point (Fig. 3b). Furthermore, ELS participants rated their sadness as higher than did non-ELS participants at the rest period after sad memory recall (VAS7 and VAS9).

#### Time-series of amygdala activity

Group (ELS and non-ELS) × Time repeated measures ANOVA showed significant main effects for time for both amygdala, left amygdala, Greenhouse-Geisser *F* (4.58, 96.01) = 3.18, *p* = .013, *η*_p_^2^ = .13, and right amygdala, *F* (8, 168) = 3.63, *p* < .001, *η*_p_^2^ = .15, and a non-significant interaction for the right amygdala, *F* (8, 168) = 1.95, *p* = .055, *η*_p_^2^ = .09. As with the time course of sadness, for associations of amygdala over time, ELS participants had similar left and right amygdala activity, VAS3-VAS9, *r*s > .70, *p*s < .08. Right amygdala activation in ELS participants was significantly or almost significantly higher after sad memory recall (VAS6 and VAS8) and during the rest period (VAS7) than in non-ELS participants (Fig. 3c). Non-ELS participants had higher activity on the left, slightly diminishing the significance of the group difference after sad memory recall during the rest period, VAS6-VAS9, *p*s > .177, *d*s < .60.

### Moderation Effects of Amygdala Reactivity on the Relationship between ELS and Symptom severity

#### Depression

**Fig. 4:**
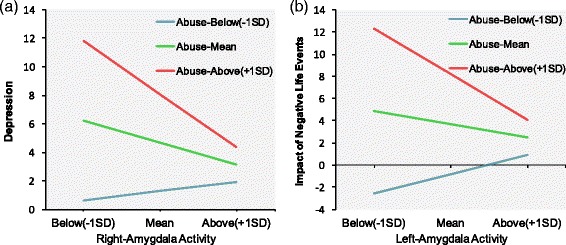
Moderation effects of amygdala reactivity. **a** Slope of the relation between right amygdala activity and depression as a function of ELS. **b** Slope of the relation between left amygdala activity and impact of negative life events as a function of ELS.

There was a significant ELS interaction with the left amygdala, *ΔF* (1, 20) = 5.43, *ΔR* ^2^ = .20, *p* = .030, *β* = -.60, and right amygdala, *ΔF* (1, 20) = 6.71, *ΔR* ^2^ = .23, *p* = .018, *β* = -.72. Results remained significant when age and behavioral differences including the degree of difficulty in recalling memories and vividness of memory recall were covaried out. As shown in Fig. 4a, left and right high amygdala activity were associated with low predicted depression scores only for the highest ELS individuals (+1SD).

#### Impact of negative stress events

There was a significant ELS interaction with the left amygdala, *ΔF* (1, 20) = 12.03, *ΔR* ^2^ = .32, *p* = .002, *β* = -.75, and right amygdala, *ΔF* (1, 20) = 4.77, *ΔR* ^2^ = .17, *p* = .041, *β* = -.61. As shown in Fig. 4b, left and right high amygdala activity predicted low impact of negative life events only for the highest (+1SD) ELS scorers.

#### Functional Connectivity of Amygdala with Other Brain Areas

**Fig. 5:**
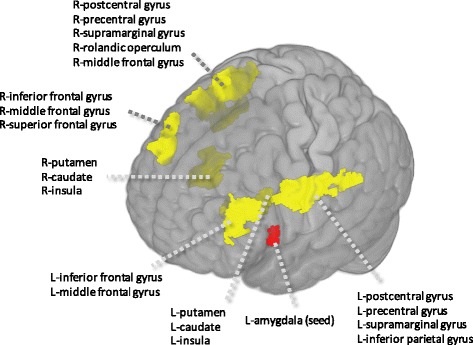
Functional connectivity map of left amygdala during sad memory recall

As summarized in Table 2 and Fig. 5, left amygdala activity in ELS people was mainly accompanied by increased functional interactions with bilateral DLPFC, bilateral motor cortex, and bilateral striatum. Associations of the right amygdala with these areas were of similar magnitude but were not significant after correction for multiple comparisons.

**Table 2:** Left amygdala functional connectivity in early life stress group during sad memory recall

Region	Location of centroid voxel	Brodmann areas	x	y	z	size	*Z*
L prefrontal cortex	L inferior frontal gyrus	46, 45, 10	-50	32	24	427	4.47
L motor cortex	L postcentral gyrus	6, 4, 40, 9, 3	-52	-8	36	551	4.45
R striatum	R putamen	-	26	16	0	427	4.45
R mortor cortex	R postcentral gyrus	6, 2, 3, 4, 40, 43, 1, 41, 13, 9, 22	66	-16	28	1086	4.45
R prefrontal cortex	R middle frontal gyrus	9, 10, 46	24	42	32	428	4.22
R striatum	L putamen	-	-22	4	6	295	4.12

*L* left, *R* right

*Note*: Threshold was set at uncorrected *p* < 0.005 for the volume of the whole brain, minimum extent 290 voxels, and at cluster levels of corrected *p* < .05

## Discussion

We examined the effects of ELS and amygdala reactivity to mood challenge on symptoms and the impact of negative life events in healthy participants screened for current and past psychiatric conditions. Negative mood induction successfully elicited amygdala activation in addition to increased sadness and decreased happiness. Consistent with the prevailing literature [49], ELS was associated with increased and sustained amygdala and sad mood reactivity to the negative mood induction, even after controlling for potential confounds such as an influence of recent negative life events. This observation could reflect increased emotional reactivity, possibly as a result of adaptations to early stress in this group [49].

That said, ELS participants in our sample had low levels of ELS. Their greater mood changes might be in contrast to individuals with higher levels of ELS, who were not measured, but in whom more blunted affect has often observed [50]. And higher amygdala reactivity was associated with decreased effects of ELS on depressive symptoms. These data suggest that while ELS may increase amygdala and emotional reactivity, this outcome may reflect a more adaptive response than the alternative – having low preserved amygdala reactivity, in never-depressed people.

Preserved robust amygdala reactivity could reflect automatic reactivity or more of an effortful engagement process. During sad memory recall, the more the left amygdala activated, the more purported regulatory areas such as the bilateral DLPFC [3], motor cortex [51], and striatum [52] also activated in individuals with abuse history. Considering that patients with a major depressive disorder showed the reduced connectivity of the left amygdala with the cortical regions linked to top-down regulation [53], these data could suggest that preserved amygdala activity in healthy individuals reflects a willful or effortful engagement with emotional material, where a less regulated individual would have a more blunted- or dampened-affect presentation.

Thus we have speculated on a potential protective effect of preserved amygdala activation in resilient adults with ELS. These data could indicate that preserved reactivity in response to early life stress, may indicate increased ability to react to and process emotional information, which may be more adaptive than blunting strategies such as shutting down or avoiding. Increased amygdala activity was generally apparent for individuals with an ELS history. This pattern of increased reactivity in response to stress is well documented in both animal and human literature [54] and has been observed to precede more detrimental apathetic reactions that occur once an individual has given up hope, e.g., as in learned helplessness/hopelessness [55, 56]. While increased amygdala activity following early stress could be beneficial for people who are resilient to depression, as compared to a more blunted style, the same pattern could be problematic for depressed people, e.g., as it is associated with rumination in depression [57]. Therefore, accounting for a history of ELS and amygdala reactivity may be useful in helping to understand and promote resilience. In individuals with a history of ELS, prior to development of disorder it may be useful to work to increase reactivity to emotional information to increase resilience. For example, techniques such as compassion meditation, which is designed to enhance compassionate feelings, can increase amygdala response to negative images [58]. In this study, increased amygdala activation was correlated with decreased depression scores in the compassion meditation group composed of healthy adults, which suggests that in some cases, increased amygdala reactivity may also be beneficial for oneself.

This study had a number of limitations. We used a self-report measure to evaluate ELS. Given observed relationships between current mood and memory recall bias [59], future studies may benefit from a prospective analysis of the relationship between ELS and subsequent changes in amygdala activity, or at least, interview based measures of childhood maltreatment or documented cases of ELS. The sample size of this study was relatively small and there were moderate, if non-significant differences between groups on multiple demographic and clinical variables. Future studies could benefit from larger and more diverse sample sizes to confirm relationships between ELS and potentially protective effects of amygdala activity, above and beyond other clinical and demographic features. Moreover, all our participants were healthy subjects with no history of depression to exclude the confounding factor of psychiatric history on ELS. To substantiate the protective role of the amygdala, future studies should include participants with a similar ELS history, but also with a history of depression as a control group. Finally, scores for ELS in this study were in a range described by our measure’s authors as the mild to moderate range. Thus, we cannot extend our inferences to higher levels of ELS.

## Conclusions

In conclusion, our results suggest that 1) ELS leads to increased amygdala reactivity in healthy people in adulthood, and 2) this reactivity could be a protective factor for depression and recent negative stressful events. If these findings were replicated with an appropriate control group, interventions to increase amygdala activity in individuals with a history of ELS may be useful for prevention of depression.

## Abbreviations

CATS: Child abuse and trauma scale
ELS: Early life stress
LES: Life event stress scale
PHQ-9: Patient health questionnaire-9
VAS: Visual analog scale
